# Association between childhood obesity and use of regular medications in the UK: longitudinal cohort study of children aged 5–11 years

**DOI:** 10.1136/bmjopen-2014-007373

**Published:** 2015-05-30

**Authors:** Francesca Solmi, Stephen Morris

**Affiliations:** Department of Applied Health Research, University College London, London, UK

**Keywords:** EPIDEMIOLOGY, PAEDIATRICS, PUBLIC HEALTH

## Abstract

**Objectives:**

Increasing rates of childhood obesity have been suggested as a possible cause for the increasing prevalence of chronic conditions among adults and children. Few studies have examined whether obese children are more likely to use medications than normal weight children. We investigate this association in the UK.

**Design:**

A panel study with repeated observations at ages 5, 7 and 11.

**Setting:**

A general population sample drawn from the Millennium Cohort Study, a UK-based birth cohort.

**Participants:**

A sample of 9667 children.

**Primary and secondary outcome measures:**

Our primary outcomes were crude and adjusted probabilities of taking any regular medications and the number of medications among overweight and obese children compared with normal weight children. Our secondary outcome was the distribution of medication use by therapeutic classification across body mass index (BMI) groups.

**Results:**

Obese children were more likely to use any medication (marginal effect (ME)=0.02, 95% CI 0.01 to 0.03) and to use more medications (ME=0.08, 95% CI 0.04 to 0.12) than normal weight children. Obese children used more medications for respiratory conditions than those of other BMI groups.

**Conclusions:**

Obese children are more likely to use regular medications and have comorbid conditions, even at young ages. This suggests that the cost of prescriptions should be considered when evaluating the economic burden of childhood obesity and that preventative strategies to reduce childhood obesity could be cost-effective in the short as well as in the long term. While more research is needed, both clinicians and policymakers should be aware of these findings when planning prevention and treatment strategies.

**Strengths and limitations of this study**

- To the best of our knowledge this is the first study investigating the association between overweight and obesity in children and use of regular medications.
- Our outcome measure was based on the point prevalence of self-reported use of regular medications, which could overestimate or underestimate the overall prevalence of medication use in the sample. We were also unable to measure use of over-the-counter medications. Our sample was underpowered to detect differences in use of medications by therapeutic classification.
- We employed a large longitudinal sample of children, which allowed us to determine patterns of medication use across time in different body mass index groups as well as type of medications used. Our study provides insight into conditions which could be comorbid with obesity in childhood.

## Background

The past 30 years have seen a rise in the prevalence of childhood obesity. Changing patterns of nutrition and physical activity, and their interplay with genetic risk factors could be the main causes of this change.^1^ In England it was estimated that, in 2012, 27.9% of children aged 2–15 years were overweight or obese compared with 25% in 1995, with a peak of 34.3% in 2004.^2^

Childhood obesity is known to persist into adulthood and to be a risk factor for several long-term health problems.^3^ Evidence suggests it is associated with cardiometabolic (diabetes, hypertension, ischaemic heart disease, stroke), respiratory (asthma), gynaecological (polycystic ovary syndrome),^4^ and musculoskeletal conditions later in life, as well as physical disability, poor mental health^5^ and cancer.^4^ ^5^ Although the greatest part of the obesity-related burden of disease is borne by adults (most disorders associated with obesity take several years to develop), in recent years conditions such as hypertension, musculoskeletal problems, type 2 diabetes, sleep apnoea and asthma have become more common among children,^6^ suggesting that higher rates of childhood obesity could be a risk factor for early onset of chronic conditions.

The association between obesity in childhood and adolescence and healthcare use and costs has been examined in primary studies^7–9^ and reviews^10–12^ with mixed findings. Most studies found that overweight and obese children incurred higher healthcare utilisation and direct costs across different healthcare settings and user groups.^9^ ^13–22^ Others did not find any differences^23^ ^24^ or found higher costs only in females^25^ or for primary care consultations.^26^ Differences in study designs, sample sizes, care settings, age groups, and healthcare systems make between-study comparisons difficult. By excluding studies not including a control group^27–29^ and not providing detailed information on cost components,^21^ ^25^ most studies examined hospital utilisation and costs,^7^ ^8^ ^14^ ^16–18^ ^20^ ^22^ ^23^ ^26^ and primary care consultations.^7^ ^8^ ^13^ ^15–20^ ^23^ ^24^ Few included pharmacy costs within total healthcare costs^15^ or investigated the use of medications^9^ ^14^ or prescriptions costs^9^ ^13^ ^14^ ^22^ and thereby reflect a trend, also seen in adult-based studies,^30^ ^31^ which potentially leads to an underestimation of obesity-associated healthcare costs.

To date, evidence on higher medication use in overweight and obese children compared with children of normal body mass index (BMI) is mixed, with some studies finding differences only in adolescents.^9^ ^22^ Two North American studies found that being overweight or obese was associated with higher costs^22^ and use^9^ of prescribed drugs among children and adolescents aged 12–19 years. An Israeli study found that, while on average there were no differences between the number of normal weight and obese children taking medications, obese children aged 4–7 years, as well as adolescents aged 12–18 years used a higher number of drugs than normal weight children.^14^ Similarly, an Australian study found that children who were obese at age 4–5 had higher prescription expenditures over 5 years.^13^

Little is also known about differences in prescribed medication use by therapeutic classification across BMI groups. One study among children in Denmark, the UK and Italy found that drugs for skin, infectious, and respiratory conditions were the most commonly prescribed in childhood.^32^ On the other hand, medications for the treatment of respiratory conditions,^9^ ^20^ as well as those for nervous conditions^9^ and diabetes,^20^ have been found to be common in overweight or obese children. The paucity of studies investigating type of medication use among the obese limits our ability to draw conclusive evidence from existing research.

To date, no UK-based study has investigated the association between medication use and obesity among children. Therefore, the aim of this study was to examine this association in children aged 5–11 years in the UK using data from the Millennium Cohort Study (MCS).

## Methods

### Sample

The MCS is a longitudinal cohort of children living in the UK at 9 months of age, born between 1 September 2000 and 31 August 2001 (for England and Wales), and 24 November 2000 and 11 January 2002 (for Scotland and Northern Ireland), who were eligible to receive Child Benefits, which at the time of sampling was a universal benefit payable to families who were permanent UK residents.^33^ The study includes children living in non-household situations and who were not born in the UK (but were residing in the UK at recruitment) and, employed a stratified clustered framework to ensure appropriate representation of the disadvantaged and ethnic minority groups.^33^ The initial recruited sample consisted of 18 552 families (72% response rate) and 18 818 children, including 246 twins and 10 triplets.^33^ This was supplemented by another sample of 692 eligible families (50% response rate) at MCS2, the second sweep of data collection which took place in 2004/2005 when the children were on average 3 years old, and this resulted in a total sample of 19 244 families and 19 517 children. More details on sampling and follow-up of the children can be found on the study's website (http://www.cls.ioe.ac.uk).

This study used data from the third (MCS3, 2006), fourth (MCS4, 2008) and fifth (MCS5, 2012) sweeps of data collection, which took place when the children were on average, 5, 7 and 11 years old, respectively. A total of 15 246, 13 857 and 13 287 families and of 15 460, 14 042 and 13 469 children were included at MCS3, MCS4, and MCS5, respectively. At MCS3, MCS4 and MCS5, 13 251 (85.7%), 11 939 (85.0%) and 11 402 (84.6%) children had complete data on all variables employed in this study. Children from Black African and Caribbean, Asian and other ethnic backgrounds, those whose main respondent had the lowest education levels or foreign qualifications, and those with lower income were more likely to have incomplete data at every sweep (see online supplementary table S1). Boys and children whose mother was overweight or obese at birth were less likely to have complete data at MCS3 and MCS5, respectively.

A total of 9667 children with complete observations over the three time-points were included in our final sample. As a sensitivity analysis, we reran our models by including children who had data for at least one of the three sweeps of data collection.

### Exposure and outcome measures

At each wave, every child's BMI was calculated from objective height and weight measurements and categorised as ‘normal (including underweight)’, ‘overweight’, and ‘obese’ using the International Obesity Task Force age-specified and gender-specific BMI cut-offs.^34^ The survey's main respondent was asked if the study child was currently taking any regular medications, defined as a medication prescribed by a doctor or a hospital (excluding over-the-counter medications), and whether these were being taken every day for 2 weeks or longer. For each child, the question was asked up to seven times in order to allow for multiple medications. From these answers we created a binary variable, indicating whether the child was taking at least one medication (1=yes, 0 otherwise), and a continuous variable indicating the total number of medications taken by the child (including 0). For each medication, their therapeutic classification was also recorded and grouped according to the British National Formulary for Children (BNFC)^i^ chapter codes. A summary binary variable was created to indicate whether the child was taking at least one medication from each therapeutic classification.

At each wave, the main respondent was asked whether the child had one or more longstanding illnesses and, if so, of which types (in MCS3 and MCS4 only). Type of longstanding illness was coded using International Classification of Diseases, Tenth Revision (ICD-10) codes; from these we derived broad families of conditions (infections, neoplasms, disorders of the blood, endocrine system, nervous system, eye, ear, circulatory system, respiratory system, digestive system, skin, musculoskeletal system and genitourinary system, mental health disorders, malformation, clinical abnormalities, injuries or poisoning, or other disorders).

### Sociodemographic and socioeconomic covariates

A number of variables (described as time-invariant if constant across time-points and time-variant if varying at each time-point) were used in descriptive analyses and as covariates in multivariate analyses.

Time-invariant variables were: child age at MCS3; gender; ethnicity (white, Black African or Caribbean, Asian—Indian, Pakistani, Bangladeshi or other Asian, mixed, and other backgrounds); main respondent's (natural mother for 99.9% of children at MCS1) highest academic qualification at MCS1 (no qualification; General Certificate of Secondary Education (GCSE)—obtained at age 16, or A levels—obtained at age 17–18; diploma of higher education; degree or higher; and any foreign qualifications); natural mother's BMI at MCS1 (pregnant; underweight, normal weight, obese). The last variable was included based on evidence of a positive association between maternal and child BMI,^35^ and as maternal obesity is a risk factor for chronic illness in the child.^36^ ^37^ Since data on natural mother's BMI was not available at MCS5, we only included values collected at MCS1 given their high correlation with those collected at MCS3 and MCS4 (r=0.87 and r=0.85, respectively).

Time-variant variables were: weekly family income equivalised using Organisation for Economic Co-operation and Development (OECD) weights (included as a continuous variable) and an indicator for sweep of data collection in order to account for secular and age-related trends.

### Data analyses

Children with complete data on exposure (BMI), primary outcomes (at least 1 medication, number of medications) and potential confounding factors for the association between BMI and medication use at MCS3, MCS4 and MCS5 (age, gender, maternal BMI, main respondent education, income) were included in the final sample. The sample with data from the three waves of data collection was described with respect to sociodemographic and socioeconomic characteristics by using cross-tabulations and analysis of variance (ANOVA).

Our primary outcomes were: (1) a binary variable indicating whether the child used at least one medication (1=yes, 0 otherwise); (2) a count variable for the total number of medications taken by the child (including 0). The first outcome was investigated with a random effects logit regression model; the second, by using a random effects negative binomial regression model. The latter was used instead of Poisson regression because our data were overdispersed (mean medications per child: 0.19, variance: 0.38).^38^ Random effects were employed under the assumption that unobserved variables were time-invariant, and within-subject variability with respect to the time-variant variables included in the model was small. Under these assumptions, SEs produced by a fixed-effect model would have been overestimated.

For both outcomes, we fitted a crude (unadjusted) and an adjusted model, the latter controlling for potential confounding factors described above. We also ran a model fitting an interaction between BMI and income, a proxy for socioeconomic status, in order to test for non-linear relationships between the two variables. We report marginal effects (ME) holding the unobserved effect at zero, showing the impact of overweight and obesity on the probability of medication use and on the number of medications used, compared with normal weight.

As secondary outcomes, we investigated the association between overweight and obesity, and type of longstanding illness in the child and medication used by doing cross-tabulations and χ^2^ tests on pooled data from MCS3 MCS4 and MCS5. Given the low numbers of longstanding illnesses and medication use in different therapeutic categories, and the lack of statistical power we did not fit regression models for individual therapeutic categories. We grouped therapeutic categories of medications according to those which obese children were more likely to use (medications for respiratory, nervous system and endocrine disorders) and unlikely to use (all the remaining categories) based on previous studies,^9^ ^20^ and ran univariate and multivariate regression models of their association with child BMI category.

The presence of longstanding illness was not included in regression models on the basis that this is on the causal pathway between obesity and medication use. Longstanding illnesses among parents were also not included for similar reasons, as they could be associated, via genetic risk factors, to longstanding illnesses in the child.

All analyses were run using Stata V.13.^39^

## Results

### Sample characteristics

A total of 9667 children were included in the final model. The majority of children were of white ethnicity and had a main respondent educated up to GCSE or A level standard or higher (table 1). More children from lower socioeconomic status groups (ie, lower income and lower levels of main respondent's education) as well as children belonging to an ethnic minority had incomplete data at all three waves of data collection; more children whose mother was overweight had incomplete data at MCS4 (see online supplementary table S1).

**Table 1:** Sociodemographic, socioeconomic, biological and health-related differences across BMI groups for all three sweeps of MCS (MCS3, MCS4 and MCS5) combined

Variables	Total N (%)	MCS			
		BMI			p (χ^2^)
		Normal (or underweight) N (%)	Overweight N (%)	Obese N (%)	
Total	29 001 (100)	22 624 (78.1)	4801 (16.5)	1576 (5.4)	N/A
Gender					
Male	14 496 (49.9)	11 593 (51.2)	2163 (45.1)	740 (47.0)	<0.0001
Female	14 505 (50.1)	11 031 (48.8)	2638 (54.9)	836 (53.0)	
Ethnicity					
White	25 287 (87.2)	19 831 (87.7)	4163 (86.7)	1293 (82.0)	<0.0001
Mixed	727 (2.5)	529 (2.3)	131 (2.7)	57 (3.6)	
Black African/Caribbean	579 (2.0)	347 (1.5)	155 (3.2)	77 (4.9)	
Asian	2049 (7.1)	1619 (7.2)	300 (6.3)	130 (8.3)	
Other	369 (1.2)	298 (1.3)	52 (1.1)	19 (1.2)	
Main respondent's highest education (MCS1)					
None	3951 (13.6)	2988 (13.2)	675 (14.0)	288 (18.3)	<0.0001
GCSE/A levels	15 777 (54.4)	12 069 (53.4)	2786 (58.0)	922 (58.5)	
Diploma higher education	2895 (10.0)	2272 (10.0)	473 (10.0)	150 (9.5)	
Degree or higher	5754 (19.8)	4805 (21.2)	784 (16.3)	165 (10.4)	
Foreign qualification	624 (2.2)	490 (2.2)	83 (1.7)	51 (3.2)	
Natural mother's BMI (MCS1)					
Pregnant	1311 (4.5)	1038 (4.6)	220 (4.6)	53 (3.4)	<0.0001
Underweight	1077 (3.7)	973 (4.3)	89 (1.9)	15 (0.9)	
Normal weight	15 840 (54.6)	13 283 (58.7)	2054 (42.8)	503 (31.9)	
Overweight	7158 (24.7)	5114 (22.6)	1542 (32.1)	502 (31.8)	
Obese	3615 (12.5)	2216 (9.8)	896 (18.7)	503 (32.0)	
Child currently taking at least one regular medication					
No	25 443 (87.7)	19 972 (88.3)	4175 (87.0)	1296 (82.2)	<0.0001
Yes	3558 (12.3)	2652 (11.7)	626 (13.0)	280 (17.8)	
Number of medicines					
0	25 443 (87.7)	19 972 (88.3)	4181 (87.0)	1296 (82.2)	<0.0001
1	2178 (7.5)	1651 (7.2)	372 (7.7)	155 (9.8)	
2	918 (3.2)	662 (2.9)	177 (3.6)	79 (5.0)	
3	309 (1.0)	233 (1.0)	48 (1.0)	28 (1.8)	
4	84 (0.3)	59 (0.3)	15 (0.3)	10 (0.6)	
5	35 (0.1)	23 (0.1)	8 (0.2)	4 (0.3)	
6	24 (0.1)	17 (0.1)	4 (0.1)	3 (0.2)	
7	10 (0.1)	7 (0.1)	2 (0.1)	1 (0.1)	
Longstanding illness					
No	24 148 (83.3)	18 887 (83.5)	4033 (84.0)	1228 (78.0)	<0.0001
Yes	4853 (16.7)	3737 (16.5)	768 (15.0)	348 (22.0)	
Sweep					
MCS3	9667 (33.3)	7718 (34.1)	1473 (30.7)	476 (30.2)	<0.0001
MCS4	9667 (33.3)	7819 (34.6)	1337 (27,9)	511 (32.4)	
MCS5	9667 (33.3)	7087 (31.3)	1991 (41.5)	589 (37.4)	

	Mean (SD)	Normal Mean (SD)	Overweight Mean (SD)	Obese Mean (SD)	p (F)
Age at entry	4.8 (0.4)	4.8 (0.4)	4.8 (0.4)	4.8 (0.4)	0.3
OECD equivalised weekly family income (MCS3–MCS4–MCS5)	352.5 (200.1)	359.0 (203.8)	337.0 (186.9)	308.0 (174.7)	<0.0001
Number of medications	0.20 (0.62)	0.18 (0.60)	0.21 (0.64)	0.31 (0.79)	<0.0001

BMI, body mass index; GCSE, General Certificate of Secondary Education; MCS, Millennium Cohort Study; N/A, not applicable; OECD, Organisation for Economic Co-operation and Development.

In MCS3, MCS4 and MCS5, 15.2%, 13.8% and 20.6% of children were overweight and 5.0%, 5.3% and 6.1% were obese, respectively (table 2). Our longitudinal sample consisted of 29 001 observations of whom 16.5% and 5.4% corresponded to a time-point in which the child was overweight or obese, respectively (table 1).

**Table 2:** Proportion of children with normal, overweight and obese BMI at each of the three waves of data collection (N=9667, MCS3, MCS4, MCS5) and distribution of medication use (using at least 1 medication) in the whole longitudinal sample (N=29 001, MCS3, MCS4, MCS5) and by BMI group

At least one prescription	MCS3 N (%)	MCS4 N (%)	MCS5 N (%)	Average medication use N (%)	Any medication use N (%)	Percentage of medication use at each time-point
Overall	9667 (100)	9667 (100)	9667 (100)			
No	–	–	–	25 443 (87.8)	9311 (96.3)	91.1
Yes	–	–	–	3558 (12.2)	2294 (23.7)	51.7
Normal weight	7718 (79.8)	7819 (80.9)	7087 (73.3)			
No	–	–	–	19 972 (88.3)	8146 (95.4)	92.4
Yes	–	–	–	2652 (11.7)	1809 (21.2)	55.6
Overweight	1473 (15.2)	1337 (13.8)	1991 (20.6)			
No	–	–	–	4175 (87.0)	2693 (90.0)	96.4
Yes	–	–	–	626 (13.0)	509 (17.0)	78.4
Obese	475 (5.0)	511 (5.3)	589 (6.1)			
No	–	–	–	1296 (82.2)	792 (87.7)	94.7
Yes	–	–	–	280 (17.8)	198 (22.0)	77.2

BMI, body mass index; MCS, Millennium Cohort Study.

More girls than boys were overweight and obese. Children of black, Asian and mixed ethnic backgrounds were also more likely to be obese, as were: children whose main respondent had no educational qualifications or qualifications up to GCSEs or A levels; children whose mother was overweight or obese at MCS1; and children who had at least one longstanding illness (table 1).

Overall medication use was 12.2% (table 2). A total of 11.7%, 13.0% and 17.8% of children in the normal weight, overweight and obese groups, respectively, took a medication, of whom 55.6%, 78.4% and 77.2%, respectively, did so at all three waves (table 2). Mean number of medications used was 0.18 (SD 0.6) for children of normal BMI, 0.21 (SD 0.6) for overweight children, and 0.31 (SD 0.8) for obese children (table 1).

The prevalence of longstanding illness was highest in children who were obese (22.0%; table 1). At age 5(MCS3), obese children had higher proportions of endocrine, respiratory, and mental health conditions as well as problems with the digestive system and clinical complications. At age 7, the most prevalent conditions among children who were obese were respiratory conditions and neoplasms (see online supplementary table S3). Respiratory conditions were the most prevalent at both ages among obese children (12.3% at age 5 and 11.5% at age 7) compared with normal weight children (7.4% at age 5 and 6.8% at age 7).

### Association between BMI category and medication use

In crude regression models (table 3), children who were overweight (ME=0.006, 95% CI 0.001 to 0.011) and obese (ME=0.023, 95% CI 0.011 to 0.035) were more likely to take at least one medication and to take a higher number of medications (overweight=ME=0.033, 95% CI 0.012 to 0.053; obese=ME=0.100, 95% CI 0.057 to 0.143) compared with children who were of normal weight. In adjusted models, obese children remained more likely to take at least one medication (ME=0.016, 95% CI 0.005 to 0.027) and to take a higher number of medications (ME=0.075, 95% CI 0.035 to 0.115). No differences were found between overweight and normal weight children in the likelihood of taking at least one medication, although a strong association was found with respect to overweight children taking more medications than normal weight children (ME=0.021, 95% CI 0.001 to 0.041). In multivariate analyses, we found some evidence of an independent association between maternal obesity and increased use of medication, and strong evidence for an association between high levels of main respondent's education, and lower use of medications and number of medications (table 3). Results were similar when we reran our models by including children who had data for at least one sweep of data collection (see online supplementary table S2). None of the interactions between income and BMI group was significantly different to zero in any of the models, and including the interaction terms did not change the size or the significance of the coefficients of any of the other variables included in the analyses (detailed results not shown).

**Table 3:** Association between overweight and obese BMI (vs normal BMI) and use of medications

	Outcome			
	Taking ≥1 regular prescriptions	Number of regular prescriptions	≥1 High-risk medication†	≥1 Low-risk medication‡
BMI group	Crude ME (95% CI)	Crude ME (95% CI)	Crude ME (95% CI)	Crude ME (95% CI)
BMI				
Normal weight	Ref.	Ref.	Ref.	Ref.
Overweight	0.006 (0.001 to 0.011)**	0.033 (0.012 to 0.053)**	0.002 (0.001 to 0.004)**	0.001 (0.001 to 0.003)
Obese	0.023 (0.011 to 0.035)**	0.100 (0.057 to 0.143)**	0.010 (0.005 to 0.016)**	−0.001 (−0.003 to 0.003)

	Adjusted ME§ (95% CI)	Adjusted ME§ (95% CI)	Adjusted ME§ (95% CI)	Adjusted ME§ (95% CI)

BMI				
Normal weight	Ref.	Ref.	Ref.	Ref.
Overweight	0.002 (−0.002 to 0.007)	0.021 (0.001 to 0.041)**	0.001 (−0.001 to 0.003)	0.001 (−0.001 to 0.003)
Obese	0.016 (0.005 to 0.027)**	0.075 (0.035 to 0.115)**	0.008 (0.003 to 0.013)**	−0.001 (0.003 to 0.02)
Age at first sweep				
4	Ref.	Ref.	Ref	Ref
5	0.002 (−0.004 to 0.008)	0.008 (−0.017 to 0.033)	0.001 (−0.001 to 0.003)	−0.001 (−0.002 to 0.001)
6	−0.02 (−0.047 to 0.011)	−0.101 (−0.258 to 0.057)	−0.004 (−0.017 to 0.007)	0.001 (−0.020 to 0.020)
Sweep				
MCS3	Ref.	Ref.	Ref	Ref
MCS4	0.011 (0.008 to 0.014)**	0.054 (0.042 to 0.067)**	0.002 (0.001 to 0.003)**	0.001 (−0.001 to 0.002)
MCS5	0.022 (0.014 to 0.031)**	0.089 (0.076 to 0.104)**	0.011 (0.007 to 0.015)**	0.002 (0.001 to 0.003)**
Maternal BMI				
Pregnant	Ref.	Ref.	Ref.	Ref.
Underweight	0.009 (−0.008 to 0.026)	0.054 (−0.026 to 0.133)	0.004 (−0.003 to 0.011)	−0.001 (−0.004 to 0.003)
Normal weight	−0.002 (−0.012 to 0.008)	−0.016 (−0.065 to 0.033)	−0.001 (−0.006 to 0.003)	0.001 (−0.001 to 0.004)
Overweight	0.002 (−0.009 to 0.013)	0.016 (−0.036 to 0.067)	0.001 (−0.004 to 0.005)	0.002 (−0.001 to 0.005)
Obese	0.013 (−0.001 to 0.026)*	0.047 (−0.011 to 0.105)	0.005 (−0.001 to 0.010)*	0.001 (−0.002 to 0.004)*
Gender				
Male	Ref.	Ref.	Ref.	Ref.
Female	−0.012 (−0.017 to −0.008)**	−0.060 (−0.081 to −0.040)**	−0.007 (−0.009 to −0.005)**	0.001 (−0.001 to 0.002)
Education				
None	Ref.	Ref.	Ref.	Ref.
GCSE/A level	−0.005 (−0.013 to 0.003)	−0.012 (−0.045 to 0.021)	−0.004 (−0.007 to −0.001)**	0.002 (0.001 to 0.004)**
Diploma higher education	−0.003 (−0.013 to 0.009)	−0.009 (−0.054 to 0.036)	−0.002 (−0.006 to 0.002)	0.002 (−0.001 to 0.005)
Degree or higher	−0.012 (−0.019 to −0.003)**	−0.030 (−0.068 to 0.008)**	−0.004 (−0.008 to −0.001)**	0.001 (−0.002 to 0.002)
Foreign qualification	−0.004 (−0.021 to 0.019)	−0.029 (−0.100 to 0.041)	−0.006 (−0.011 to −0.001)**	0.001 (−0.004 to 0.004)
Ethnicity				
White	Ref.	Ref.	Ref.	Ref.
Mixed	0.017 (−0.003 to 0.036)	0.056 (−0.026 to 0.140)	0.002 (−0.005 to 0.008)	0.002 (−0.003 to 0.007)
Black African/Caribbean	0.001 (−0.015 to 0.016)	0.009 (−0.066 to 0.084)	−0.001 (−0.006 to 0.006)	−0.001 (−0.005 to 0.004)
Asian	0.007 (−0.004 to 0.016)	0.002 (−0.038 to 0.043)	−0.001 (−0.004 to 0.002)	0.002 (−0.001 to 0.006)
Other	0.001 (−0.019 to 0.019)	−0.041 (−0.115 to 0.033)	−0.002 (−0.009 to 0.005)	−0.002 (−0.007 to 0.003)
OECD equivalised income	−0.001 (−0.001 to 0.001)	−0.001 (−0.001 to 0.001)	−0.001 (−0.001 to 0.001)*	0.001 (−0.001 to 0.001)

**p<0.05; *0.1>p>0.05.

†High-risk medications: medications for respiratory, central nervous system and endocrine conditions.

‡Low-risk medications: medications for gastrointestinal, cardiovascular, infections, obstetric, malignant, nutrition and blood, musculoskeletal, ear, eye and skin conditions.

^§^Adjusted: age, gender, ethnicity, main respondent education, OECD equivalised income, natural mother's BMI.

BMI, body mass index; GCSE, General Certificate of Secondary Education; MCS, Millennium Cohort Study; ME, marginal effect; OECD, Organisation for Economic Co-operation and Development.

### Association between BMI category and medication use by therapeutic classification

Medications for respiratory (5.6%), skin (1.1%) and central nervous system (0.8%) disorders were most commonly used (table 4). Use of medications from other therapeutic categories was low (all <0.8%). Overweight and obese children had greater use of medications for respiratory conditions and overweight children had greater use of medications to treat infections. Obesity, but not overweight, had a strong association with greater use of medications for respiratory, endocrine and central nervous system disorders combined (ME=0.008, 95% CI 0.003 to 0.013), but not for all the other conditions combined (table 3). These results were unchanged after including an interaction term between BMI and income, which was non-significant (results not shown).

**Table 4:** Medication use by therapeutic category and BMI category

Variables	Total N (%)	MCS3–MCS5			
		BMI			p Value
		Normal N (%)	Overweight N (%)	Obese N (%)	
Gastrointestinal					
None	28 841 (99.4)	22 503 (99.5)	4773 (99.4)	1565 (99.3)	0.6
At least one	160 (0.6)	121 (0.5)	28 (0.6)	11 (0.7)	
Cardiovascular					
None	28 986 (99.9)	22 613 (99.9)	4797 (99.9)	1576 (100.0)	0.4
At least one	15 (0.1)	11 (0.1)	4 (0.1)	0 (0.0)	
Respiratory					
None	27 383 (94.4)	21 450 (94.8)	4507 (93.9)	1426 (90.5)	<0.0001
At least one	1618 (5.6)	1174 (5.2)	294 (6.1)	150 (9.5)	
Central nervous system					
None	28 784 (99.2)	22 454 (99.2)	4769 (99.3)	1561 (99.0)	0.5
At least one	217 (0.8)	170 (0.8)	32 (0.7)	15 (1.0)	
Infections					
None	28 920 (99.7)	22 567 (99.8)	4779 (99.5)	1574 (99.9)	**0**.**02**
At least one	81 (0.3)	57 (0.2)	22 (0.5)	2 (0.1)	
Endocrine					
None	28 896 (99.6)	22 550 (99.7)	4780 (99.6)	1534 (99.4)	0.09
At least one	105 (0.4)	74 (0.3)	21 (0.4)	10 (0.6)	
Obstetrics					
None	28 972 (99.9)	22 602 (99.9)	4797 (99.9)	1573 (99.4)	0.5
At least one	29 (0.1)	22 (0.1)	4 (0.1)	3 (0.2)	
Malignant disease					
None	28 994 (99.9)	22 621 (99.9)	4798 (99.9)	1575 (99.94)	0.08
At least one	7 (0.02)	3 (0.01)	3 (0.1)	1 (0.06)	
Nutrition and blood					
None	28 946 (99.8)	22 580 (99.8)	4793 (99.8)	1573 (99.8)	0.9
At least one	55 (0.2)	44 (0.2)	8 (0.2)	3 (0.2)	
Musculoskeletal					
None	28 986 (99.9)	22 613 (99.9)	4797 (99.9)	1576 (100.0)	0.4
At least one	15 (0.1)	11 (0.1)	4 (0.1)	0 (0.0)	
Eye					
None	28 990 (99.9)	22 615 (99.96)	4799 (99.96)	1576 (100.0)	0.7
At least one	11 (0.1)	9 (0.04)	2 (0.04)	0 (0.0)	
Ear–nose					
None	28 918 (99.7)	22 563 (99.7)	4785 (99.7)	1570 (99.6)	0.6
At least one	83 (0.3)	61 (0.3)	16 (0.3)	6 (0.4)	
Skin					
None	28 687 (98.9)	22 381 (98.9)	4748 (98.9)	1558 (98.1)	0.9
At least one	314 (1.1)	243 (1.1)	53 (1.1)	18 (1.1)	

Total numbers of medications do not add up to those in table 3 due to missing values on the type of medication variable.

BMI, body mass index; MCS, Millennium Cohort Study.

## Discussion

We investigated the association between overweight and obesity, and medication use in children aged 5–11 years. We found that, overall, 12.2% of children used prescribed medications regularly, although children who were obese had a higher probability of using at least one regular medication and a higher number of medications compared with children of normal BMI. We also found that overweight and obese children were more likely to use medications for respiratory, endocrine and central nervous system diseases, which are potentially related to obesity.

Research on the use of prescription drugs in children is scarce and differences in study methodologies make direct comparisons difficult. To our knowledge, only one study has addressed prevalence of medication use at this age in the UK,^32^ but it did not investigate whether there were differences across BMI categories. We found that obese and, to a lesser extent, overweight children had a higher probability of using prescription drugs. Our findings are consistent with some non-UK studies,^8^ ^27^ but not others.^9^ ^14^ Differences in exposure definition, study design and sample size may account for any discrepancies. Kuhle *et al*^9^ found that overweight or obese Canadian adolescents, but not children, had a higher prescription drug use than those who were normal weight. We found an effect in obese, but not overweight children, and it is possible that combining overweight and obese BMI categories may have diluted the effect in the Canadian study. This study also employed a cross-sectional design, which could underestimate medication use compared with a repeated observation design, since obese children were likely to use medications at all time-points in MCS. An Israel-based study found no variation in use of medications between children in different obesity groups, but it did find a higher number of medication used by obese children compared with normal weight children.^14^ Although this study assessed medication use over a longer time period and more accurately through the use of medical records, the much smaller sample size (363 obese children, 382 normal weight children) could have resulted in type II error, explaining the lack of a significant association between obesity and medication use.

Obesity is associated with several long-term conditions which usually occur later in life^3^ and it has been shown that English adults who are obese are more likely to use prescription drugs for cardiovascular, gastrointestinal, respiratory, central nervous system and endocrine conditions, and gynaecological and musculoskeletal disorders.^31^ Recent evidence suggests that the prevalence of some of these conditions in children is rising as a consequence of obesity.^6^ By observing higher use of medications for specific therapeutic classifications of drugs in children who were obese, our findings appear to provide some evidence in support of these findings. We found a strong association between childhood obesity and use of any medication for respiratory, endocrine and central nervous system conditions, which have been previously identified as ‘at risk’ therapeutic categories for obese children.^9^ ^20^ Based on simple associations, this finding may be driven predominantly by an association between obesity and use of medications for disorders of the respiratory system; notably, we found that almost twice as many obese children compared with normal weight ones used medications for respiratory conditions, which has been previously documented.^9^ The positive association between asthma and obesity in childhood is one which has received growing attention in recent years and good evidence exists on the association between the two although causal patterns remain unclear.^40^ ^41^ Our findings suggest that obese children are more likely to suffer from comorbid respiratory conditions and thus, incur higher healthcare expenditures. A better understanding of the aetiology of this comorbidity is warranted to devise cost-effective policies for the prevention and management of these conditions in childhood.

The presence of a socioeconomic gradient in the distribution of childhood obesity has been previously documented in the literature.^42^ ^43^ In our sample, a greater proportion of children who were obese had lower family income, lower levels of main respondent's education, and belonged to ethnic minorities. This reflected in both lower ME for the association between child BMI and use of medications, once these factors were accounted for in multivariate analyses, and in an independent association between lower parental education and higher use of medications. Although we were not able to discriminate in this study as to whether a change in trend has occurred over time, a previous study using data from the Health Survey for England has shown that, while the prevalence of childhood obesity in England had been reduced during the years in which MCS had also taken place, its socioeconomic gradient had not^42^ and our findings seem to confirm this result. Since children from low socioeconomic status appear to be more vulnerable to obesity, policies and educational programmes aimed at reducing lifestyle risk factors for obesity (such as sedentary lifestyle and poor nutrition) targeting families with low level of literacy are warranted to reduce both the short-term and long-term health and economic impact of childhood obesity.

We also found an independent association between maternal obesity and higher use of medications in the child. This suggests that maternal obesity could signal morbidity in the mother, which could be passed on to the child by means of genetic predisposition. However, we also found an association between maternal and child obesity, confirming previous findings lending support to the hypothesis of an intergenerational transmission of obesity.^35^ ^44^ ^45^ Since both childhood obesity and maternal BMI emerged as strong predictors of medication use in childhood policies aimed at curbing obesity in the child could have the potential for lowering the risk in children who might be genetically predisposed to this disease.

This study has several strengths. First, it is the first to investigate the association between obesity and medication use in childhood in the UK. We used a large representative longitudinal sample of children that was followed over three time-points at ages 5, 7 and 11. We were, therefore, able to determine patterns of medication use across time in different BMI groups. As well as employing child's use of any medications as outcome in our analyses, we were also able to investigate the number and type of medications used, which provided some insight on which conditions might be more frequently comorbid with obesity in childhood. Finally, we were able to adjust for other variables, which could have potentially confounded the association being investigated.

Nevertheless, some limitations also need to be acknowledged. This study relies on point prevalence of self-reported medication use by the main household respondent which could overestimate or underestimate the prevalence of our outcome and, thus, of its association with the exposure under investigation. The study also only included prescribed medications taken by the child every day for at least 2 weeks, which could result in an underestimation of total medication use in the sample, for example, by not including over-the-counter medications and by requiring use every day for at least 2 weeks. Our sample was underpowered to detect differences in use of medications by therapeutic classification, given the low use of some types of medication in this age group. However, we were able to group medications that are likely to be related to obesity. More research using primary and secondary care registers and a longer time series is warranted in order to provide more accurate estimates. We found selective attrition with respect to children from low socioeconomic status at all waves of data collection. Since we found some evidence of a gradient in socioeconomic status and both obesity and medication use this means that we could be underestimating this association in our study. We included underweight in our normal BMI category; this could lead to an underestimation of the association with medication use as underweight children might be more likely to be ill. We attempted to use a first-differences approach, regressing change in taking medication between sweeps on change in BMI group, but the majority of children (>85%) were classified as either normal weight or overweight/obese in all three sweeps. We could not employ survey weights given the panel structure of the data; therefore, some of the effects of confounding variables, such as ethnicity, on the association between exposure and outcome could have been missed in our analyses. Finally, in our study we evaluated associations only, and were not able to identify causal effect of obesity on medication use. For example, it may be that medication use affects obesity or that there are unobserved factors, such as time preference, that affect both medication use and obesity. Further research to identify causal effects, for example, using instrumental variable regression techniques, would be beneficial.

In conclusion, obese children in the UK are more likely to use prescribed medications confirming that these costs should be considered when evaluating the cost of childhood obesity. Our findings also suggest that even at young ages, obesity is associated with a number of comorbid conditions, with some evidence of a socioeconomic gradient in this association. More research aimed at capturing both intergenerational and environmental risk factors for obesity and medication use as well as healthcare use and its associated costs in children is needed as a starting point for devising cost-effective prevention strategies, and analyses investigating the costs of childhood obesity should include the costs of medication use as well as other healthcare costs.

## Footnotes

i The BNFC (http://www.bnf.org) is a reference manual providing advice to healthcare professionals about prescribing, dispensing, monitoring and administering medicines to children. It has chapters categorising medications into the therapeutic classifications employed in this study—01: gastrointestinal system; 02: cardiovascular system; 03: respiratory system; 04: central nervous system; 05: infections; 06: endocrine system; 07: obstetrics, gynaecology & urinary tract disorders; 08: malignant disease and immunosuppression; 09: nutrition and blood; 10: musculoskeletal and joint diseases; 11: eye; 12: ear, nose and oropharynx; 13: skin.
